# Expression and Characterization of a New PolyG-Specific Alginate Lyase From Marine Bacterium *Microbulbifer* sp. Q7

**DOI:** 10.3389/fmicb.2018.02894

**Published:** 2018-11-29

**Authors:** Min Yang, Yuan Yu, Suxiao Yang, Xiaohui Shi, Haijin Mou, Li Li

**Affiliations:** College of Food Science and Engineering, Ocean University of China, Qingdao, China

**Keywords:** alginate lyase, *Microbulbifer*, heterologous expression, extracellular secretion, thermal stability, poly-guluronic acid preference

## Abstract

Alginate lyases play an important role in preparation of alginate oligosaccharides. Although a large number of alginate lyases have been characterized, reports on directional preparation of alginate oligosaccharides by alginate lyases are still rather less. Here, a gene *alyM* encoding a new alginate lyase AlyM was cloned from *Microbulbifer* sp. Q7 and expressed in *Escherichia coli*. AlyM exhibited the maximumactivity at pH 7.0 and 55°C and showed special preference to poly-guluronic acid (polyG). Glycine promoted the extracellular secretion of AlyM by 3.6 times. PBS and glycerol significantly improved the thermal stability of AlyM, the enzyme activity remained 75 and 78% after heat-treatment at 45°C for 2 h, respectively. ESI-MS analysis suggested that AlyM mainly produced oligosaccharides with degrees of polymerization (DP) of 2–5. The results of ^1^H-NMR showed that guluronic acid (G) occupied the reducing end of the end products, indicating that AlyM preferred to degrade the glycosidic bond at the G-X linkage. HPLC analysis showed that the hydrolysis products with a lower degree of polymerization contained more G. Therefore, AlyM shows good potential to produce alginate oligosaccharides with specific M/G ratio and molecular weights.

## Introduction

Alginate is a major acidic polysaccharide in brown algae, such as *Sargassum vulgare* (Sari-Chmayssem et al., 2016), *Ecklonia radiate* (Lorbeer et al., 2015), and *Turbinaria ornate* (Zubia et al., 2008); it is also found in the exopolysaccharide of several bacteria, including *Pseudomonas aeruginosa* (Mccaslin et al., 2015). Alginate is a linear polysaccharide consisting of 1 → 4 linked β-D-mannuronic acid (M) and its C-5 epimer α-L-guluronic acid (G). As a kind of gel, alginate is often used in the field of food and medical materials (Lee and Mooney, 2012). However, its high viscosity and high degree of polymerization (DP) restrict its application.

Alginate lyase present in diverse organisms (Singh et al., 2011; Wang et al., 2013), degrades alginate by β-elimination and produces unsaturated oligosaccharides with double bonds at the non-reducing end (Ma et al., 2008). Alginate degradation products have been developed for broad applications in agricultural, health product and medical industries, since they exhibit biological activities including immunomodulation, anti-tumor, antioxidant, and plant growth-promoting activities (Yokose et al., 2009; Tusi et al., 2011; Zhou et al., 2015). Two groups of alginate lyases can be classified based on the substrate specificity, one is the G block-specific lyases (polyG lyases, EC 4.2.2.11), and the other is the M block-specific lyases (polyM lyases, EC 4.2.2.3) (Kam et al., 2011). Based on the sequences similarity, alginate lyases have been assigned to the polysaccharide lyase (PL) families PL5, PL6, PL7, PL14, PL15, PL17, and PL18. In general, PL5, PL6, PL14, and PL17 families showed polyM specificity, PL7, PL15, and PL18 families showed polyM and polyG specificity (Lombard et al., 2010). Various alginate lyases have been cloned and characterized, such as AlyA1, AlyA2 and AlyA3 from *Azotobacter vinelandii*, alyA from *Pseudoalteromonas atlantica* AR06, AlyFRA and AlyFRB from *Falsirhodobacter* sp. Alg1, alyPM from *Pseudoalteromonas* sp. SM0524, Aly2 from *Flammeovirga* sp. strain MY04 (Gimmestad et al., 2009; Matsushima et al., 2013; Chen et al., 2016; Mori et al., 2016; Peng et al., 2018a). However, it is also difficult to prepare oligosaccharides directionally due to the low enzyme activity and broad substrate specificity. Therefore, it is of great importance to find new alginate lyases to meet with the industrial application.

In a previous study, we isolated a marine bacterium, *Microbulbifer* sp. Q7 from the gut of a sea cucumber which could produce alginate lyases. According to the sequencing result of the whole genome of Q7, five alginate lyase-encoding sequences were predicted (Yang et al., 2018). In this work, a gene *alyM*, encoding alginate lyase AlyM, was studied for the cloning and expression. The degradation products of AlyM were analyzed by infrared spectroscopy (IR), high performance liquid chromatography (HPLC), nuclear magnetic resonance (NMR) spectroscopy, and electrospray ionization mass spectrometry (ESI-MS).

## Materials and methods

### Strains, plasmids, and media

The alginate lyase-producing strain *Microbulbifer* sp. Q7 (= CGMCC 14061) was cultured in LB medium (10% tryptone, 5% yeast extract, and 10% NaCl). *Escherichia coli* BL21 (DE3) was cultured in LB medium. The pProEX-HTa plasmid was used as a cloning and expression vector. Alginate (M/G ratio 0.85) was purchased from Sinopharm Chemical Reagent Beijing Co., Ltd. PolyM and polyG (purity>90%) were purchased from Qingdao BZ Oligo Biotech Co. Ltd. (Qingdao, China).

### Cloning and expression of the recombinant alginate lyase

The signal peptide and restriction sites were predicted by using the SignalP 4.1 server (http://www.cbs.dtu.dk/services/SignalP/) and NEBcutter V2.0 (http://tools.neb.com/NEBcutter2/index.php), respectively. ProtParam (http://web.expasy.org/protparam/) was used to determine the identity of the protein and predict the molecular mass of the mature protein. The phylogenetic tree of the amino acid sequences of alginate lyases was built using MEGA 7.0. The homology modeling of protein structures was carried out using SWISS-MODEL (https://swissmodel.expasy.org/interactive).

One pair of primers (PF: 5′-GEGAATTCRLATGAAAGTAAGTTGCGCTGTC-3′; PR: 5′-AAGCTTRTTAATCGTGCGACTGCTCC-3′, with the *EcoRI* and *HindIII* sites in PF and PR underlined, respectively) were designed using primer premier 6 with reference to the *alyM* sequence. PCR was performed in a thermal cycler using PrimeSTAR HS DNA Polymerase (Takara Bio Inc., Japan) and Q7 genomic DNA as a template. The PCR conditions were as follows: 2 min at 98°C, followed by 30 cycles of 10 s at 98°C, 5 s at 54°C, and 2 min at 72°C, with a final extension step for 5 min at 72°C. The PCR products were sequenced by the Beijing Genomics Institute (BGI) and the completely correct sequence was digested with *EcoRI* and *HindIII* and then ligated to *EcoRI*- and *HindIII*-digested expression vector (pProEX-HTa). The recombinant plasmid containing the PCR product was designated HTa-*alyM*.

*Escherichia coli* BL21 (DE3) cells harboring HTa-*alyM* were cultivated in LB medium to produce recombinant alginate lyase. After fermentation for 24 h, the fermentation liquor was centrifuged, and the supernatant was taken as extracellular enzyme. The precipitate cells were suspended in PBS buffer (100 mM, pH 7.0), and disrupted by ultrasonication to obtain intracellular enzyme.

### Optimization of fermentation conditions

In order to improve the expression of the *alyM* gene, inducer concentration, and induction temperature were studied. To determine the optimal inducer concentration, BL21-HTa-*alyM* was pre-incubated at 37°C for 2 h, then inducer with different concentrations was added into the culture medium and cultivation continued at 23°C (IPTG) or 28°C (lactose). To determine the optimal induction temperature, the BL21-HTa-*alyM* was pre-incubated at 37°C for 2 h, then 0.9 mM IPTG or 0.4% lactose was added into the culture medium and cultivated at different temperatures. The effects of additives (0.02% SDS, 0.5% Gly, 2% Tween-80, and 2% TritonX-100) on the extracellular enzyme accumulation were also examined.

### Enzyme activity assays

The activity of recombinant enzyme, AlyM, was assayed using the 3,5-dinitrosalicylic acid method (Miller, 1959). Briefly, 100 μL of enzyme solution was mixed with 900 μL of substrate solution [0.5% (w/v) in 50 mM phosphate buffer (pH 7.0)] and incubated at 45°C for 10 min. One unit of enzyme (U) was defined as the amount of enzyme causing the release of 1 μmol of reducing sugar from alginate per minute. The protein concentration was determined by the Bradford method using bovine serum albumin as the standard (Bradford, 1976).

### Purification of recombinant alginate lyase

The extracellular enzyme was purified by Ni-NTA agarose column (Cube Biotech, Germany) which pre-equilibrated in 50 mM phosphate buffer containing 100 mM NaCl, pH 7.4 (buffer A). The AlyM was fully absorbed by Ni-NTA agarose column (1.6 × 5 cm), then His-tagged target protein was eluted using buffer A containing 10, 100, 200, and 400 mM imidazole (20 mL) in sequence. The purified AlyM was detected by 12% sodium dodecyl sulfate polyacrylamide gel electrophoresis (SDS-PAGE). The purified enzyme was dialyzed for further analysis.

### Characterization of the recombinant alginate lyase

The AlyM was reacted with 0.5% alginate, polyM, and polyG, respectively, to determine the substrate specificity. The kinetic parameters of AlyM were determined by measuring the enzyme activity using these three substrates with different concentrations (0.025–0.2 mg/mL) at 45°C, pH 7.0 for 10 min. The values of *K*_*m*_ and *V*_*max*_ were calculated by double-reciprocal Lineweaver and Burk plots.

The optimum temperature for alginate lyase activity was determined in 50 mM sodium phosphate buffer, pH 7.0, at various temperatures ranging from 40 to 60°C. The effect of pH on alginate activity was determined at optimum temperature in various buffers (50 mM) such as citrate buffer (pH 4.0–6.0), phosphate buffer (pH 6.0–8.0), and glycine-NaOH buffer (pH 9.0-10.0). The AlyM was incubated at various temperatures (35–50°C) for 6 h to determine the thermal stability. The effect of NaCl, glycerol, and PEG1000 with different concentrations on thermal stability were evaluated by incubating the enzyme at pH 7.0 and 45°C for 2 h. The thermal stability of enzyme was also measured by heat-treatment in different buffer solutions at pH 7.0 and 45°C for 2 h. After the treatment, enzyme activity was measured as the method mentioned above. The effect of metal ions and chelators on alginate lyase activity was examined by monitoring enzyme activity in the presence of various chemical reagents.

### Circular dichroism (CD) analysis

The CD spectra of AlyM and the enzyme from the original wild-type strain were analyzed using a MOS-450 circular dichroism spectrometer (Bio-logic) as described previously (Niu et al., 2015). Briefly, the flow rate of nitrogen was maintained above 5 L/min, the wavelength range was from 190 to 250 nm, the acquisition time was 0.5 s, the resolution was 1 nm. The concentration of AlyM in 20 mM phosphate buffer (pH7.4) was set at 0.5 mg/mL, the 20 mM phosphate buffer was used as the blank. The Dichroweb online software was used to estimate the percentages of secondary structures (α-helix, β-sheet, β-turns, and loops) (Whitmore and Wallace, 2008).

### Analysis of alginate lyase-degradation products

The AlyM was mixed with 2% alginate and incubated at 45°C for approximately 6 h to obtain the hydrolysis products. The hydrolysis products were separated and purified by Bio-Gel P4 Polyacrylamide Gel (Bio-Rad Laboratories, Inc. US), and each of the purified components was analyzed by ^1^H-NMR. The hydrolysis products were freeze-dried with a vacuum freezer dryer and analyzed by infrared spectroscopy (IR), negative ion ESI-MS, and nuclear magnetic resonance (NMR) spectroscopy (Jouanneau et al., 2010; Jagtap et al., 2014; Swift et al., 2014).

To determine the monosaccharide composition of hydrolysis products, the hydrolysis products were successively precipitated by ethanol with different volumes. The precipitates were freeze-dried with a vacuum freezer dryer and applied for the decomposition by 2 M TFA at 110°C for 4 h. The samples were detected by HPLC after PMP (1-phenyl-3-methyl-5-pyrazolone) pre-column derivatization (Wang et al., 2018). The analysis of HPLC using a XDB-C18 column (Agilent Technologies Inc., Santa Clara, CA, USA) under the following conditions: the mobile phase was comprised of 50 mM KH_2_PO_4_ (pH 6.9), the column temperature was 25°C, the flow velocity was 1 mL/min, and detection was achieved by the UV detector at 245 nm. The molecular weight (Mw) of the precipitates was detected by Gel Permeation Chromatography (GPC) using the column of PL aquagel-OH 30 (Agilent Technologies Inc., USA). The 200 mM NaNO_3_ with10 mM NaH_2_PO_4_ was used as mobile phase at a flow rate of 0.6 ml/min. The precipitates were dissolved in mobile phase and filtered through 0.22 μm filter. The inject volume was 20 μL.

### Statistical analysis

The results of optimization of fermentation conditions and enzymatic characterization experiments were analyzed by the standard deviation method, using SPSS 18.0 (IBM, New York, NY, USA). All original data represented three biological replicates. The data presented in tables are expressed as means ± standard deviations.

## Results

### Sequence analysis of alyM

**Figure 1:**
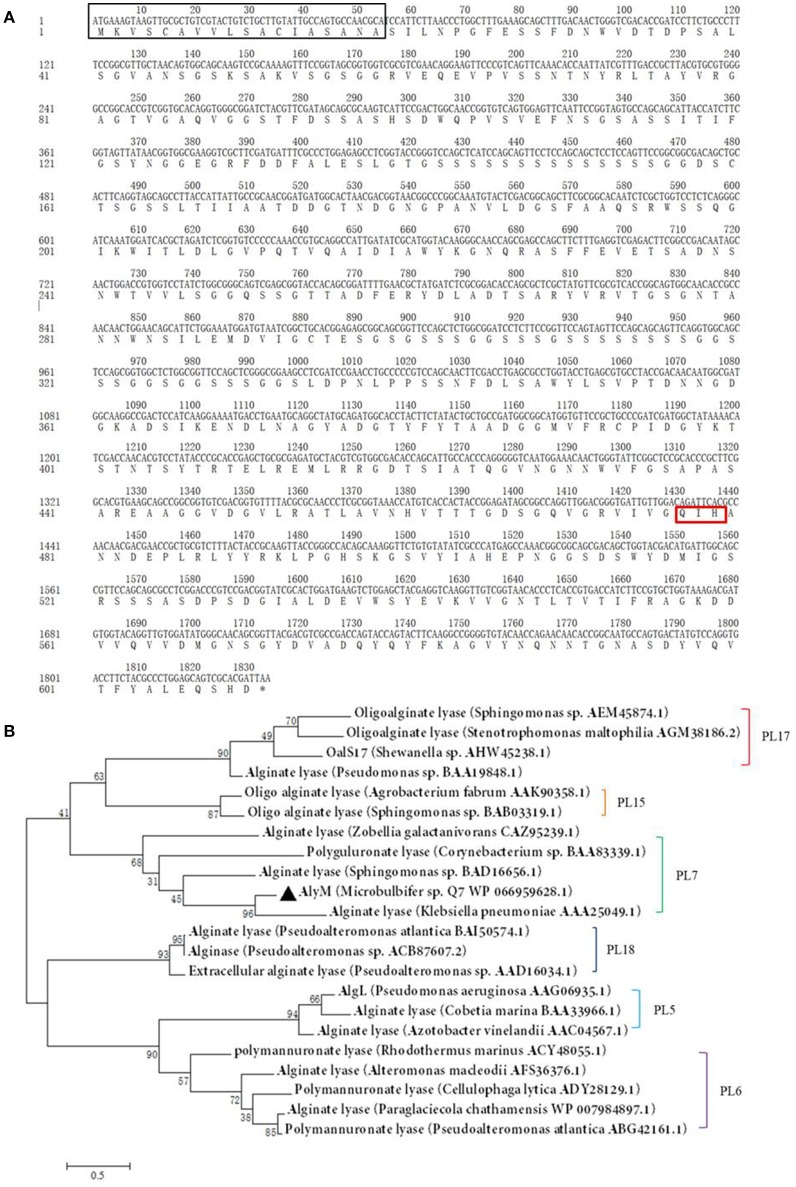
**(A)** Nucleotide sequences and deduced amino acid sequences of the alginate lyase gene from *Microbulbifer* sp. Q7. The *black box, red box* (Q^477^-I^478^-H^479^), and *asterisk* mark the signal peptide sequence, catalytic residues and stop codon, respectively. The deduced amino acid sequence contains three conserved domains: CBM_4_9 (N^17^-G^82^), F5_F8_type_C (D^177^-M^289^), and alginate_lyase2 (F^344^-H^609^) domains. **(B)** Phylogenetic analysis of AlyM. This phylogenetic tree was calculated by the method of maximum likelihood. Genbank accession numbers and organism names are given. Branch numbers indicate the bootstrap values in the ML analysis. Arcs with different colors indicate different PL families.

The nucleotide sequence of *alyM* was 1,833 bp, and the deduced amino acid sequence of AlyM was 610 aa, with an 18-aa signal peptide (Figure 1A). AlyM contained three conserved domains: CBM_4_9 (N^17^-G^82^), F5_F8_type_C (D^177^-M^289^) and alginate_lyase2 (F^344^-H^609^) domains. Among them, alginate_lyase2 domain was the catalytic domain. The 3D structure model of AlyM was built based on the template 4ozx.1.A which showed a highest quality, but the structure model only contained alginate_lyase2 domain. The structure model contained a cavity for substrate binding (Figure S1). The molecular mass (Mw) and isoelectric point (pI) of the AlyM predicted by ProtParam were 62,994 Da and 4.4, respectively. The relationship between AlyM and other characterized alginate lyases which belong to PL5, PL6, PL7, PL15, PL17, and PL18 families was reflected by phylogenetic analysis (Figure 1B). AlyM showed the highest similarity (64%) with PL7 alginate lyase (*Klebsiella pneumonia* AAA25049.1), indicating that AlyM might be a new PL7 family alginate lyase.

### Expression and fermentation optimization of recombinant alginate lyase

**Figure 2:**
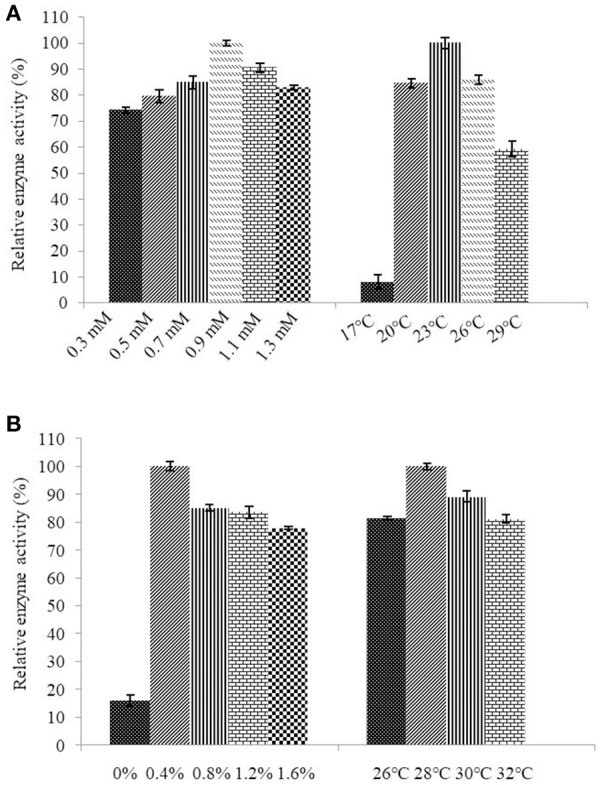
Induction of IPTG **(A)** and lactose **(B)** on the extracellular expression of AlyM. The BL21-HTa-*alyM* was pre-incubated at 37°C for 2 h, then inducer with different concentrations (IPTG: 0.3, 0.5, 0.7, 0.9, 1.1, and 1.3 mM; lactose: 0, 0.4, 0.8, 1.2, and 1.6%) was added into the culture medium and cultivation continued at 23°C (IPTG) or 28°C (lactose) to determine the optimal inducer concentration. The BL21-HTa-*alyM* was pre-incubated at 37°C for 2 h, then 0.9 mM IPTG or 0.4% lactose was added into the culture medium and cultivated at different temperatures (IPTG:17, 20, 23, 26, and 29°C; lactose: 26, 28, 30, and 32°C) to determine the optimal induction temperature.

**Figure 3:**
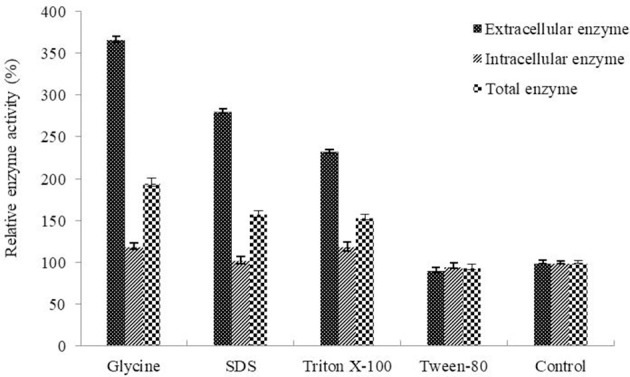
Effects of additives on the expression of AlyM. The additives included 0.5% (w/v) Gly, 0.02% (w/v) SDS, 0.2% (v/v) TritonX-100, and 2% (v/v) Tween-80. BL21-HTa-*alyM* was pre-incubated at 37°C for 2 h, then 0.4% lactose was added into the culture medium and cultivation continued at 28°C for 22 h.

Determination of the optimum induction conditions was necessary for the secretion of recombinant enzyme. As shown in Figure 2A, the optimal inducer temperature of IPTG was 23°C, the optimal IPTG concentration was 0.9 mM. The optimal conditions of lactose induction are shown in Figure 2B. The optimal inducer temperature was 28°C, the optimal lactose concentration was 0.4%. As shown in Figure 3, three additives examined in this study promoted the expression of AlyM. Compared with the control group, 0.5% Gly, 0.02% SDS, and 2% TritonX-100 increased the enzyme extracellular secretion by 3.6 times, 2.8 times, and 2.3 times, respectively. For the extracellular and total enzyme activity, Gly was chosen as the extracellular secretory inducer.

### Purification and properties of the recombinant alginate lyase

**Figure 4:**
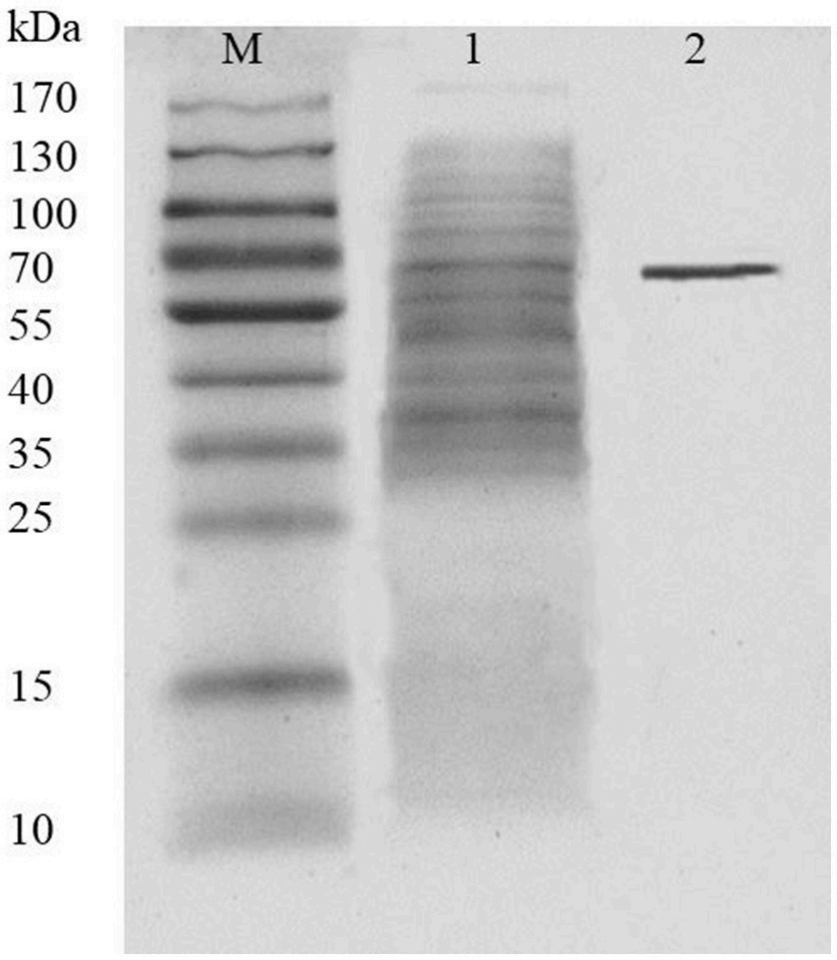
SDS-PAGE analysis of recombinant AlyM expression and purification. M, marker (Thermo Fisher Scientific Inc., Waltham, MA, USA); 1, extracellular recombinant AlyM; 2, affinity-purified recombinant AlyM.

AlyM was purified by affinity chromatography. The result of SDS-PAGE analysis of the crude recombinant protein and the affinity-purified protein indicated that the Mw of AlyM was approximately 63 kDa, which is close to the predicted Mw of 62,994 Da (Figure 4). According to the results of substrate specificity assay, AlyM showed higher activity with alginate and polyG than with polyM (Table 1). The AlyM has the maximum affinity to polyG, shown by its lowest *K*_*m*_ values toward polyG.

**Table 1:** Activities and kinetic parameters of AlyM with alginate, polyM, and polyG.

**Substrate**	**Alginate**	**PolyM**	**PolyG**
Activity (U/mL)	8.71 ± 0.79	1.28 ± 0.12	8.19 ± 0.82
*K_*m*_* (mg/mL)	0.48 ± 0.06	1.65 ± 0.31	0.37 ± 0.08
*V_*max*_* (U/mg)	74.22 ± 5.37	16.72 ± 3.21	62.49 ± 5.65

*The extracellular enzyme was mixed with substrate solution [0.5% (w/v) in 50 mM phosphate buffer (pH 7.0)] and incubated at 45°C for 10 min to determine the enzyme activity. The values of K_m_ and V_max_ were determined by measuring the enzyme activity using the three substrates (alginate, polyM, and polyG) with different concentrations (0.025, 0.05, 0.1, 0.15, and 0.2 mg/mL) at 45°C, pH 7.0 for 10 min and calculated by double-reciprocal Lineweaver and Burk plots*.

**Figure 5:**
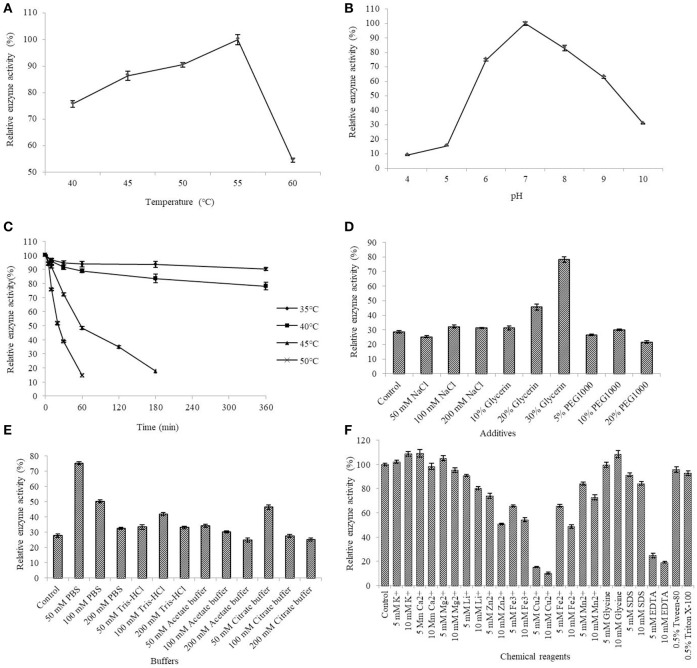
Biochemical characterizations of AlyM. **(A)** The optimal reaction temperature of the enzyme. **(B)** The optimal reaction pH of the enzyme. **(C)** Thermal stability of AlyM at pH 7.0. The purified enzyme was heat-treated at 35–50°C for 6 h and assayed for residual enzyme activity to evaluate the thermal stability. **(D)** The effect of NaCl, glycerol, and PEG1000 with different concentrations on AlyM thermal stability. The enzyme was heat-treated at 45°C for 2 h and the residual enzyme activity was measured to evaluate the thermal stability. **(E)** The effect of different buffers (PBS, Tris-HCl, acetate buffer, and citrate buffer) with different concentrations (50, 100, and 200 mM) on thermal stability of the enzyme. The enzyme was heat-treated in different buffer solutions at 45°C for 2 h and assayed for residual enzyme activity to evaluate the thermal stability. **(F)** Effects of chemical reagents on the enzyme activity. Reaction systems without addition of chemical reagents were used as control.

The AlyM activity maximum at the conditions of 55°C and pH 7.0 (Figures 5A,B). The results of the thermal stability assay showed that AlyM was stable at the temperature no more than 40°C (Figure 5C). The residual enzyme activity of AlyM was 32.8% after incubated at 45°C for 2 h, and was 14.7% after incubated at 55°C for 1 h. Compared with the control, 30% glycerol increased markedly the enzyme thermal stability (Figure 5D). As shown in Figure 5E, different buffer solutions improved enzyme thermal stability with varying degrees. The residual enzymatic activity of AlyM treated in 50 mM PBS, 100 mM Tris-HCl, 50 mM acetate buffer, and 50 mM citrate buffer were 2.7, 1.5, 1.2, and 1.7 times as that of control, respectively. The highest thermal stability of AlyM as observed in 50 mM PBS and the residual enzymatic activity was 75% after treated at 45°C for 2 h.

The effects of different additives with different constructions on enzyme activity are shown in Figure 5F. The 5 mM K^+^, 10 mM K^+^, 5 mM Ca^2+^, 5 mM Mg^2+^, and 10 mM glycine slightly enhanced AlyM activity, 5 mM Li^+^, 10 mM Li^+^, 5 mM Mn^2+^, and 10 mM SDS slightly inhabited AlyM activity, whereas 5 mM Zn^2+^, 10 mM Zn^2+^, 5 mM Cu^2+^, 10 mM Cu^2+^, 5 mM Fe^3+^, 10 mM Fe^3+^, 5 mM Fe^2+^, 10 mM Fe^2+^, 10 mM Mn^2+^, 5 mM EDTA, and 10 mM EDTA strongly inhibited AlyM activity. The presence of the emulsifiers 0.5% Tween-80 and 0.5% Triton X-100 had almost no influence on AlyM activity, as did 5 mM SDS.

### CD analysis of alyM

**Figure 6:**
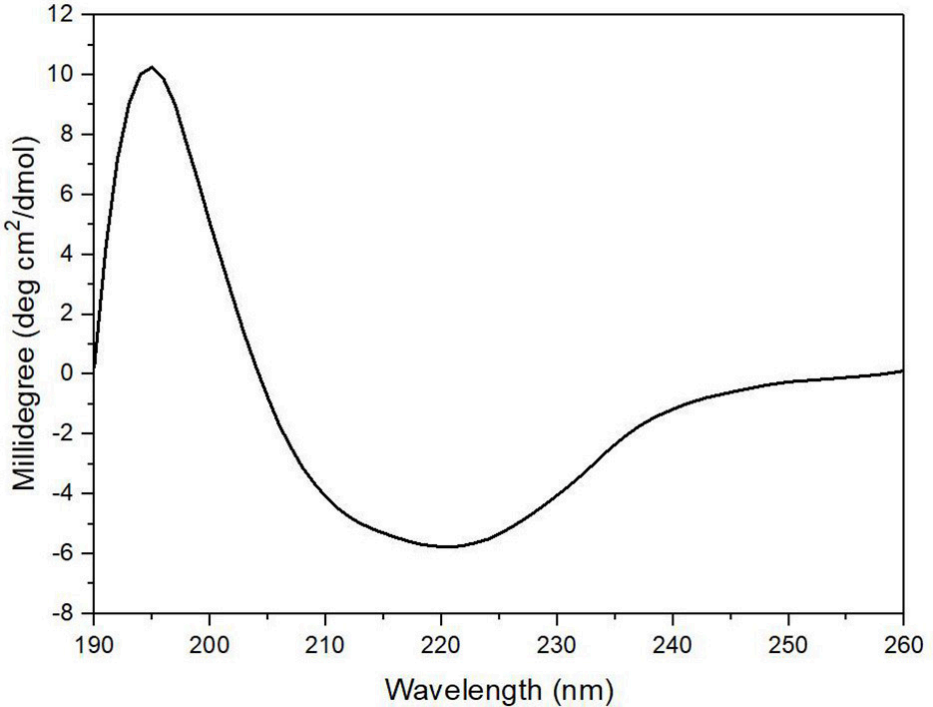
The CD spectrum of AlyM. The concentration of AlyM in 20 mM phosphate buffer (pH7.4) was set at 0.5 mg/mL, the 20 mM phosphate buffer was used as the blank.

The enzyme secondary structure was determined by CD. The alginate lyase AlyM displayed a peak at 195 nm and a valley around 220 nm (Figure 6). The results indicated that the AlyM was mainly composed of β-sheets and β-turns. The percentages of AlyM secondary structure elements helix, β-sheet, β-turn and random coil were 2.8, 49.1, 22.9, and 25.2%, respectively. In addition, the spectrum of AlyM was similar to that of the enzyme from the original wild-type strain, which means the clone did not change the enzyme structure (Figure S2).

### Analysis of hydrolysis products

**Figure 7:**
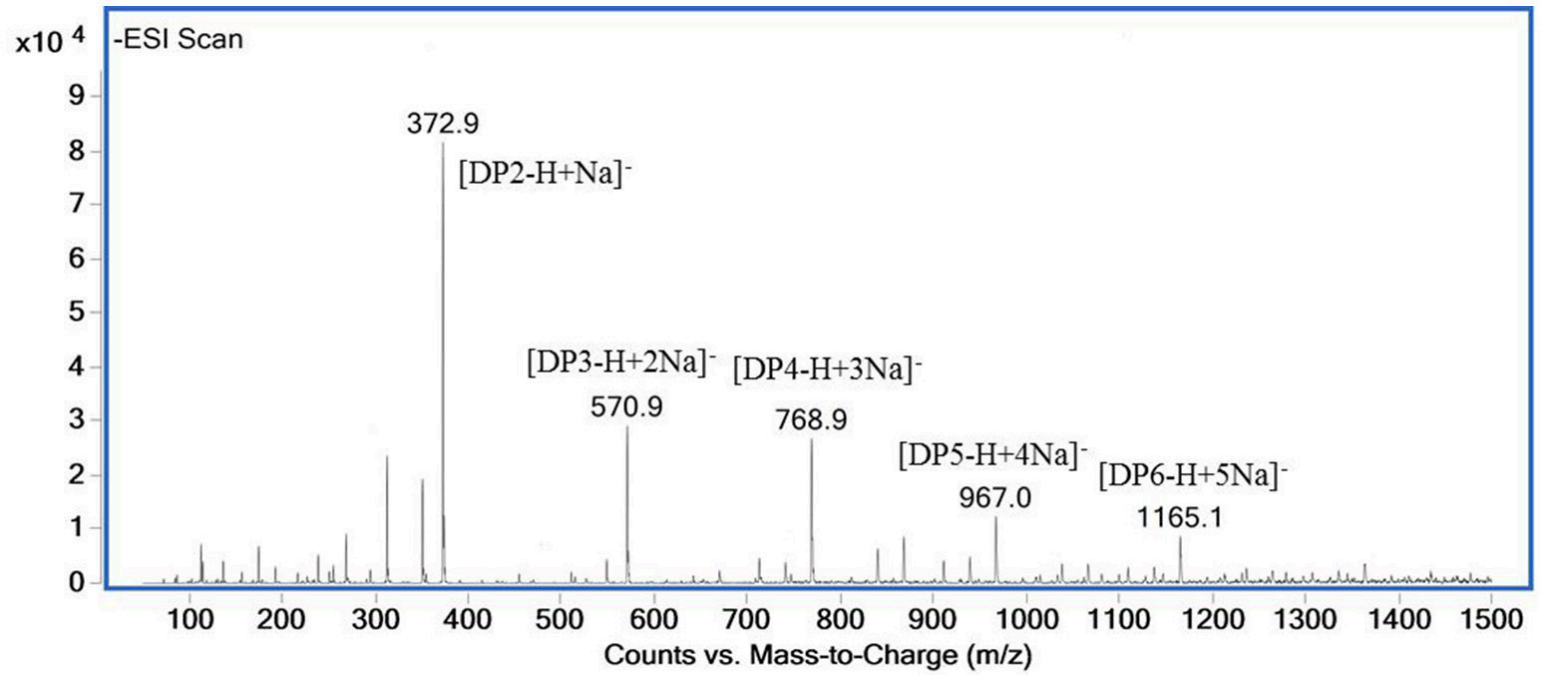
ESI-MS of the AlyM hydrolysis products.

The ESI-MS spectrum for hydrolysis products is shown in Figure 7. The ions at 373, 571, 769, 967, and m/z 1,165 represented unsaturated disaccharide (374 Da), unsaturated trisaccharide (572 Da), unsaturated tetrasaccharide (770 Da), unsaturated pentasaccharide (968 Da), and hexasaccharide (1,166 Da), respectively. It verified that AlyM cleave the glycosidic bonds in alginate by β-elimination reaction.

**Figure 8:**
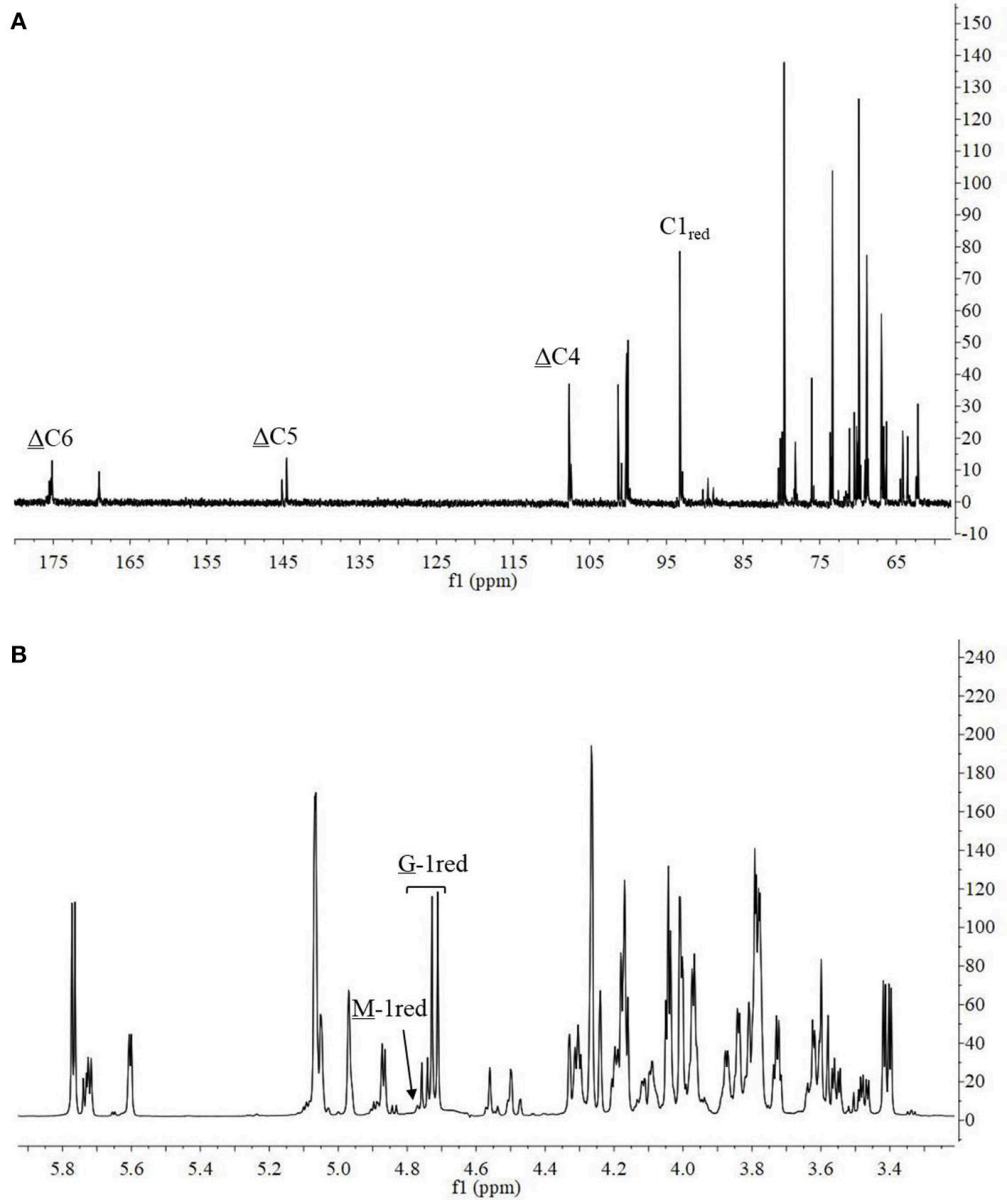
^13^C-NMR **(A)** and ^1^H-NMR **(B)** of AlyM hydrolysis products.

The ^13^C-NMR spectrum of the hydrolysis products is shown in Figure 8A. The chemical shift of the carboxyl carbon was usually 168–176 ppm (Leone et al., 2006). In this study, the C6 reducing end appeared at 175.14 ppm, and the non-reducing end appeared at 169.04 ppm. The chemical shifts of C5 and C4 at the non-reducing end appeared at 145.53 and 107.69 ppm, respectively. Chemical shift at 65–85 ppm was from C2–C5 of reducing end, and that at 93–96 ppm was from C1 (Holtan et al., 2006; Redouan et al., 2009). In ^1^H-NMR spectrum, the reducing end can be easily determined by the chemical shift of the anomeric proton. The chemical shifts at 4.70–4.75 ppm with ^3^*J*_HH_ = 8.6 Hz and 4.75–4.80 ppm with ^3^*J*_HH_ < 2 corresponded to the β-anomeric protons of the G and M residues, respectively (Marchler-Bauer et al., 2013). In Figure 8B, the chemical shift at 4.70–4.75 ppm with ^3^*J*_HH_ = 8.6 Hz was strong and the chemical shift at 4.75–4.80 ppm ^3^*J*_HH_ < 2 was weak, indicated that the reducing end was mainly G. Thus, it indicated that AlyM probably cleaved the glycosidic bond at the G-G or G-M linkage.

**Figure 9:**
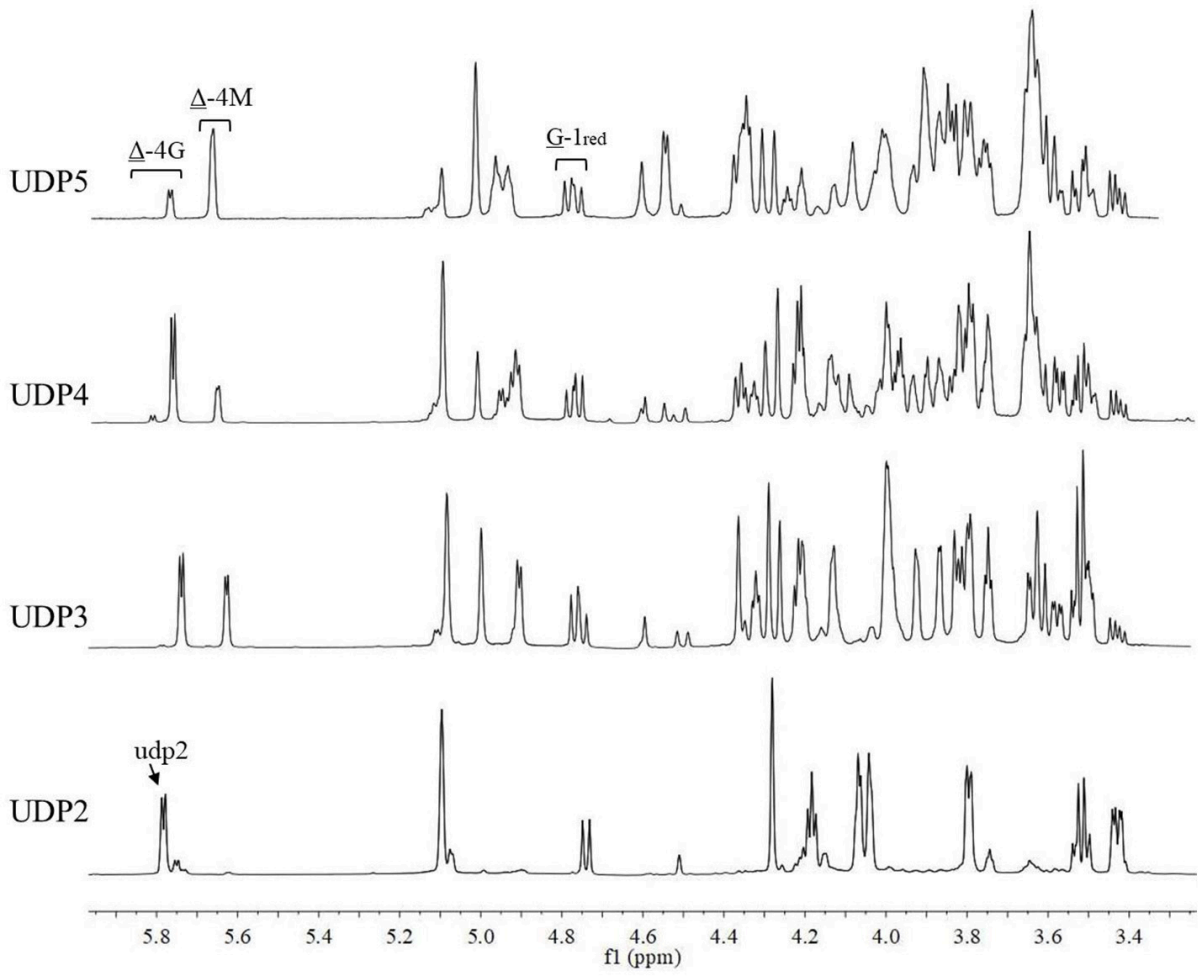
^1^H-NMR analysis of the end products of the digestion of alginate by AlyM. The fractions UDP2, UDP3, UDP4, and UDP5 were purified by Bio-Gel P4 Polyacrylamide Gel. “udp2” was the typical chemical shift of alginate oligosaccharide with UDP2.

The chemical shifts at the non-reducing end were dependent on the neighboring residue, and the neighboring residue can be identified by the chemical shift at the 4H of non-reducing end (Gimmestad et al., 2009). The chemical shift at 5.75 ppm of Δ-4H indicated that the neighbor was G residue, while the appearance of the Δ-4H signal at 5.64 ppm indicated that the neighbor was M residue. The ^1^H-NMR spectra of separated and purified end products of the digestion of alginate by AlyM are shown in Figure 9. The UDP2 fraction showed a strong signal at Δ-4G and produced almost no Δ-4M signals. It indicated that the component of UDP2 fraction was ΔG. The UDP3 fraction showed strong signals at Δ-4G and Δ-4M, and the molar ratio of ΔGX to ΔMX was 1.3. Moreover, the G-1_red_ signal was observed in the UDP3 fraction. These results demonstrated that the trisaccharides were ΔGG and ΔMG. The UDP4 fraction showed strong signals at Δ-4G and weak signals at Δ-4M; the G-1_red_ signal was also present in this ^1^H-NMR spectrum. However, the UDP5 fraction showed strong signals at Δ-4M and weak signals at Δ-4G, and the ratio was 2.9. There was no chemical shift of M-1_red_ in the purified unsaturated oligosaccharides, further indicating that AlyM cleaved the glycosidic bond at the G-X linkage.

**Figure 10:**
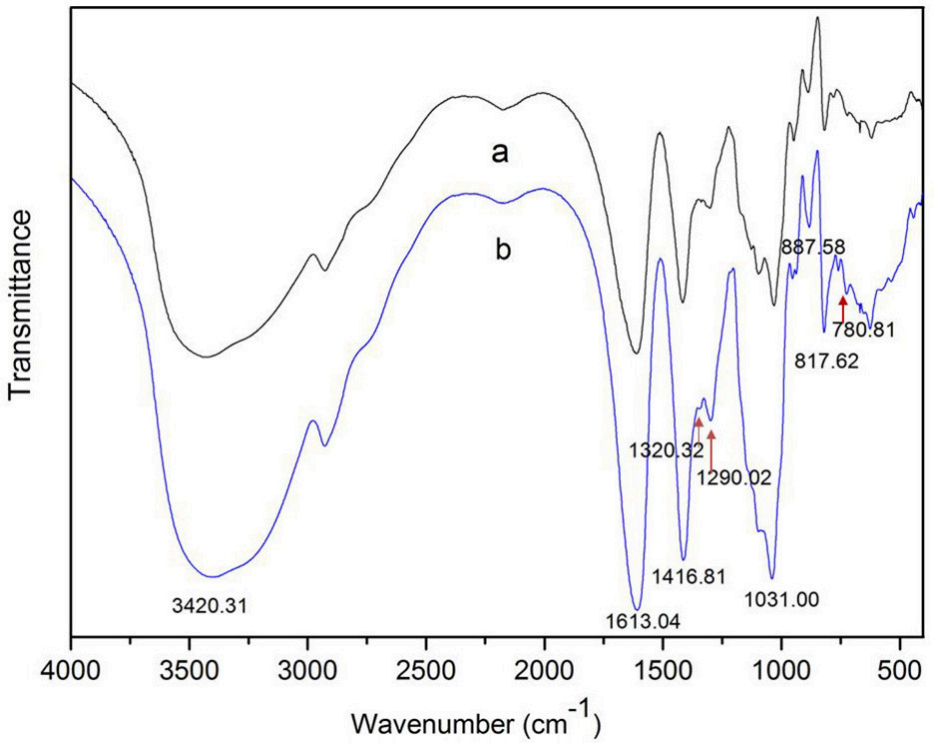
IR analyses of alginate and the AlyM hydrolysis products. **(a)**, alginate; **(b)**, hydrolysis products.

**Figure 11:**
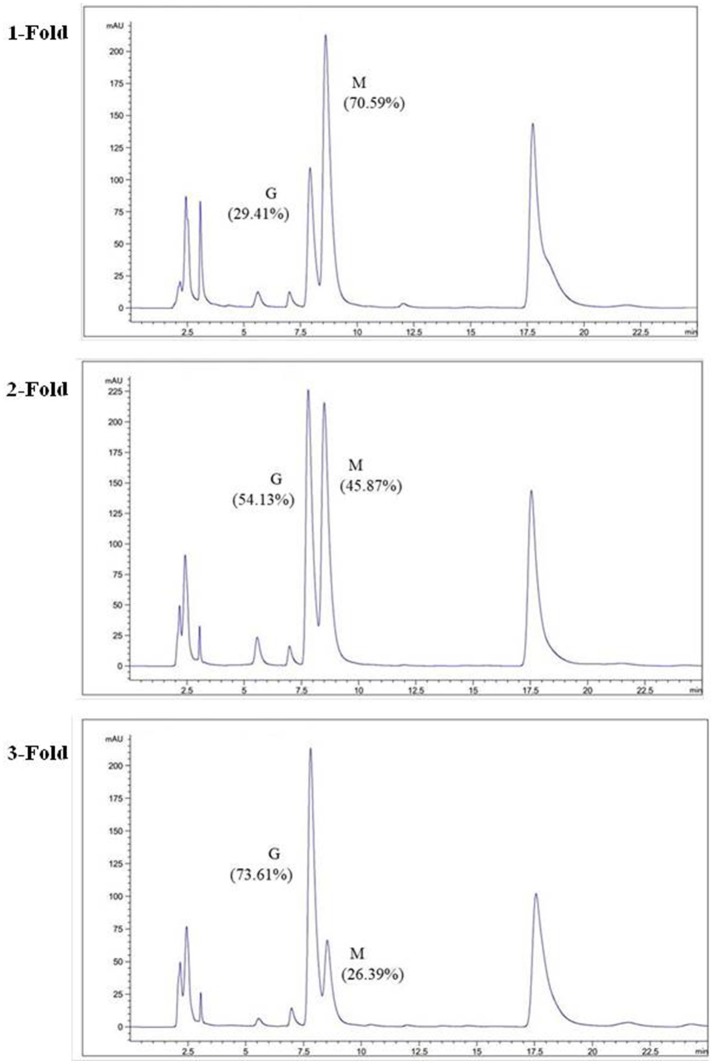
HPLC analyses of hydrolysis products precipitated by ethanol with different volumes.

The degradation products were compared to alginate using IR spectra. Based on the data shown in Figure 10, the hydrolysis products maintained the characteristics of alginate, since the side group spectra had no significant changes. In the IR spectrum, the peak at 3,420 cm^−1^ was a hydroxyl stretching vibration; the peak at 2,926 cm^−1^ was a carbonyl stretching vibration; and the peaks at 1,613 and 1,416 cm^−1^ were asymmetrical and symmetrical carboxyl stretching vibrations, respectively. The peaks at 1,290 and 1,320 cm^−1^ correlated with the M and G content, respectively (Salomonsen et al., 2008). GPC analysis showed that the Mw of hydrolysis products were 6.82, 3.14, and 1.93 kDa, respectively, after being successively precipitated by 1-, 3-, and 5-fold ethanol (data not shown). The results of HPLC of the hydrolysis products are shown in Figure 11. The peaks at 8.106 min and 8.818 min were monosaccharide standard of G and M, respectively (Sánchez-Machado et al., 2004). Based on the method of integration of the peak areas, the M/G ratios of hydrolysis products successively precipitated by 1-, 3-, and 5-fold ethanol were 2.44, 0.85, and 0.37, respectively. It meant that the hydrolysis products with a lower DP contained more G. The result was in accordance with the ^1^H-NMR spectra, which showed that AlyM cleaved the G-X linkage. Therefore, the fractions rich in G are easier to be degraded into the products with lower DP.

## Discussion

In this study, a new polyG-specific alginate lyase-encoding gene, *alyM*, from marine bacterium *Microbulbifer* sp. Q7, was cloned and expressed. The extracellular enzyme was used to investigate the enzyme properties and the optimum enzyme specific activity was 74.22 ± 5.37 U/mg. The protein contains 610 aa with a Mw of 63.0 kDa, which is near to that of AlyA5 from *Zobellia galactanivorans*, 69.5 kDa (Thomas et al., 2013). The percentages of AlyM secondary structure elements were similar to that of the enzyme from the original wild-type strain, which indicated that cloning and expression did not affect the enzyme structure. Among the reports of alginate lyases, AlyM appeared to have the maximum activity toward alginate (Table 2). The *K*_*m*_ value of AlyM was 0.48 mg/mL, which was lower than the reported, such as OalA, OalB, and OalC from *Vibrio splendidus* 12B01 (3.25, 0.76, and 0.53 mg/mL, respectively) (Jagtap et al., 2014) and Algb from *Vibrio* sp. W13 (0.67 mg/mL) (Zhu et al., 2015b), indicating that AlyM has higher affinity to alginate.

**Table 2:** Comparison of AlyM with other alginate lyases.

**Enzyme**	**Source**	**Activity (U/mg)**	***K_m_* (mg/mL)^a^**	**Cation activators**	**Cation inhibitors**	**Optimal pH**	**Optimal Temp. (°C)**	**Thermal stability (°C)^b^**	**Substrate specificity**	**Products (DP)**	**References**
AlyM	*Microbulbifer* sp. Q7	74.2	0.48	K^+^, Mg^2+^, Ca^2+^	Zn^2+^, Cu^2^, Fe^3+^, Fe^2+^	7.0	55	45	polyG	2–5	This study
OalS6	*Shewanella* sp. Kz7	33.7	0.91	Na^+^, K^+^	Zn^2+^, Cu^2+^, Ni^2+^, Al^3+^, Fe^3+^	7.2	40	45	polyG	1	Li et al., 2015
AlgMsp	*Microbulbifer* sp. 6532A	–	0.92	–	Zn^2+^, Cu^2+^, Ni^2+^, Fe^3+^, Ca^2+^	8.0	50	–	polyM	2–5	Swift et al., 2014
ALG-5	*Streptomyces* sp. ALG-5	–	–	Co^2+^, Mg^2+^, Ca^2+^, Mn^2+^	Zn^2+^, Ba^2+^	7.5	50	45	polyG	2–3	Shin et al., 2011
A1-IV	*Sphingomonas* sp. A1	0.1	–	Mn^2+^	Al^3+^, Fe^3+^, Co^2+^, Mg^2+^	8.5	37	45	–	–	Miyake et al., 2003
Alg-S5	*Exiguobacterium* sp. Alg-S5	21.8	0.91	–	–	7.5	40	50	–	–	Mohapatra, 2017
ALW1	*Microbulbifer* sp. ALW1	1.5	–	Na^+^	Ni^2+^, Co^2+^, Ba^2+^, Zn^2+^, Cu^2+^, Fe^3+^	7.0	45	45	polyG	2–3	Zhu et al., 2015c
AlgA	*Pseudomonas* sp. *E03*	40.0	–	Na^+^, K^+^, Mg^2+^, Ca^2+^, Zn^2+^, Ba^2+^	Co^2+^, Cu^2+^, Fe^3+^, Mn^2+^	8.0	30	45	polyM	2–5	Zhu et al., 2015a
OalS17	*Shewanella* sp. Kz7	34.0	–	Na^+^, K^+^	Fe^3+^, Al^3+^	6.2	50	45	polyM	1–5	Wang et al., 2015
OalC6	*Cellulophaga*sp. SY116	33.7	0.95	Na^+^, K^+^, Ca^2+^	Cu^2+^, Mn^2+^ Ni^2+^, Fe^3+^, Al^3+^, EDTA	6.6	40	40	polyG	1–2	Li et al., 2018
OalC17	*Cellulophaga*sp. SY116	36.9	0.75	Na^+^, Ca^2+^	Cu^2+^, Mn^2+^ Ni^2+^, Fe^3+^, Al^3+^, EDTA	7.8	45	35	polyM	1–2	Li et al., 2018

a *The affinity toward alginate*.

b *The thermal stability means the temperature that residual enzyme activity more than 50% after the enzyme incubated without buffers at different temperature for X min (X = 60, 60, 30 10, 30, 120, 30, 60, 60, and 60, respectively from top to bottom)*.

The optimal activities of most examined alginate lyases are present at pH 7.0–8.5, and AlyM showes optimal activity at pH 7.0, which was within this range. The optimal temperatures for alginate lyases are typically 30–50°C, whereas AlyM showes optimal activity at 55°C. However, AlyM has low thermal stability, the residual enzyme activity was only 32% after incubated at 45°C for 2 h. As a protective agent, glycerol was reported to improve the stability of enzyme as hydroxyl content increasing (Wu et al., 2011). It is consistent with the result in this study, the AlyM shows increasing thermal stability in the presence of 30% glycerol. However, the addition of glycerol increased the viscosity of the enzyme solution, making the enzyme unfavorable for its application. The enzymatic reaction was usually carried out in buffer solution to prevent the instability of enzyme resulted from the pH fluctuation during the reaction. In this work, PBS, Tris-HCl, and citrate buffer could protect the enzyme from inactivation during the heat-treatment. Among them, PBS showed the most effective protection, keeping 75% residual enzyme activity. In previous studies, phosphate enhanced the thermal stability of green fluorescent protein at pH 7.0 (de Lencastre Novaes et al., 2015) and phosphate ions reduced the denaturation of bovine serum albumin at an acidic pH (Xu and Grassian, 2017). It was speculated the structure and function of proteins could be regulated by salt ions (Formaneck et al., 2006). Several heavy metal ions such as Zn^2+^, Cu^2+^, Fe^2+^, Fe^3+^, and Mn^2+^ strongly inhibited enzyme activity. As shown in the deduced amino acid sequence, AlyM contains five cysteines (Cys), among which Cys^393^ is located in the catalytic domain. It was reported that heavy metal ions such as Zn^2+^, Cu^2+^, Fe^2+^, Fe^3+^, Pb^2+^, Mn^2+^, and Cd^2+^ have strong affinities for sulphydryl (-SH) residues (Yao et al., 2012). The presence of heavy metal ions results in the negative conformational change of enzyme, because the combination of heavy metal ions and sulfhydryl groups affects the chemical bonds and nucleophilicity of the protein (Blundell and Jenkins, 1977; Yu et al., 2017). It indicated that a free sulfhydryl group is necessary for AlyM exerting enzyme activity or maintaining integrity of protein conformation. The metal ions such as Mg^2+^ and Ca^2+^ can maintain the active conformation of the enzyme to prevent it denaturation (Dou et al., 2013). The importance of Mg^2+^ and Ca^2+^ for some alginate lyases from marine bacteria has also been reported (Inoue et al., 2016; Li et al., 2018).

IPTG is a commonly used inducer which is not metabolized by bacteria, but it is potentially toxic to human body (Mahoney, 1998; Chaudhary and Lee, 2015). Compared with IPTG, lactose is a safe, non-toxic inducer that can be used as a carbon source by bacteria, but its induction effect is far less than that of IPTG (Tan et al., 2012). In this study, the induction effect of IPTG and lactose was compared, the extracellular enzyme activity induced by lactose (2.42 U/mL) was higher than that of induced by IPTG (1.96 U/mL). Previous studies showed that glycine increased the extracellular secretion of recombinant enzymes due to it enhanced the permeability of both outer and inner membrane (Li et al., 2012). In this study, compared with the control group, glycine increased the extracellular enzyme activity and total enzyme activity by 3.6 and 1.9 times, respectively. Lactose induction and glycine supplementary make the alginate lyase more effective for the development of alginate oligosaccharides.

Alginate lyases with high substrate specificity are beneficial for the preparation of alginate oligosaccharides with specific structures. The general consensus is that the Mw and M/G ratio of oligosaccharides are important factors controlling their functional activities. When comparing alginate oligosaccharides with the same molecular weights, higher M/G alginate oligosaccharides exhibited better DPPH∙ scavenging activity (Sen, 2011). While the alginate oligosaccharides with lower M/G ratio showed a higher glyceollin-inducing activity (Peng et al., 2018b). The high guluronic acid-containing alginate oligosaccharide exhibited higher efficiency of anti-pathogens and anti-fungal activities (Powell et al., 2013; Tondervik et al., 2014). In addition, high guluronic acid-containing alginate oligosaccharides reduced the LPS-stimulated inflammatory and osteosarcoma progression and improved the ability of antioxidant and anti-inflammatory (Zhou et al., 2015; Chen et al., 2017). In the present study, AlyM degraded alginate to produce high guluronic acid-containing alginate oligosaccharides. These alginate oligosaccharides can be used in many fields as antibacterial, anti-inflammatory, and anti-tumor compounds.

In conclusion, we report the cloning, expression in *E. coli*, and characterization of a new PL7 alginate lyase of *Microbulbifer* sp. Q7. The AlyM showed higher activity than the alginate lyases reported before. PBS and glycerol significantly increased recombinant enzymes' thermal stability at 45 °C. The identified AlyM preferably degraded the glycosidic bond at the G-X linkage in alginate. In addition, dimers, trimers, tetramers and pentamers with guluronic acid as the reducing end are the main end-products after adequate hydrolysis. Furthermore, AlyM catalyzes to yield alginate oligosaccharides with a specific M/G ratio and molecular weight, showing good potential for the development of functional alginate oligosaccharides in the food and medicine industry.

## Author contributions

MY performed all the experiments, coordinated the data analysis, and prepared this manuscript. HM and LL contributed in the experimental proposal and manuscript polishing. YY, SY, and XS prepared part of research materials.

### Conflict of interest statement

The authors declare that the research was conducted in the absence of any commercial or financial relationships that could be construed as a potential conflict of interest.
