# Supplementary material for: Expression and Characterization of a New PolyG-Specific Alginate Lyase From Marine Bacterium Microbulbifer sp. Q7
Source: Front Microbiol. 2018 Nov 29;9:2894. doi: 10.3389/fmicb.2018.02894 (PMC6281962; doi:10.3389/fmicb.2018.02894)
Supplement: Supplementary file 1 [file Table_1.DOCX]

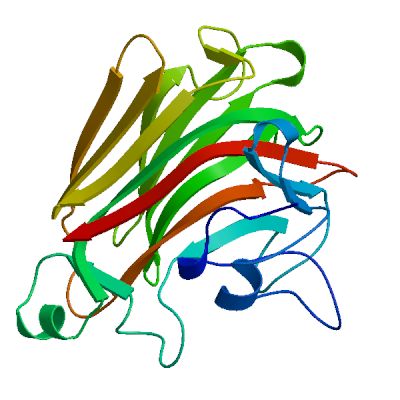


**FIGURE S1** Homology modeling of AlyM (residues 338-610)


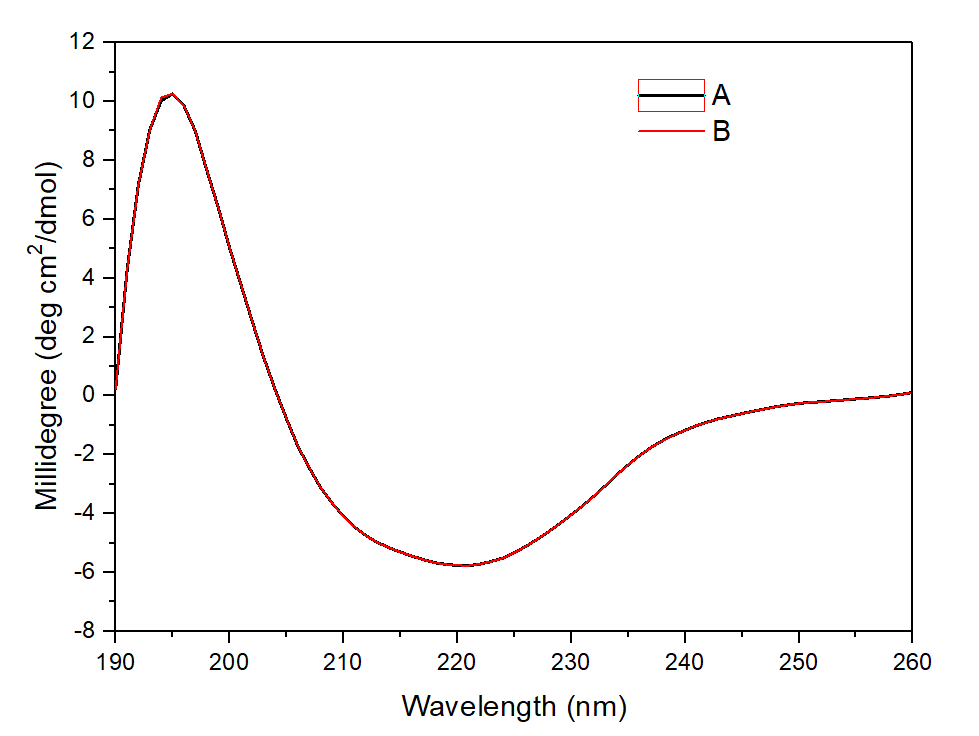


**FIGURE S2** The CD spectra of AlyM (A) and the enzyme from the original wild-type strain (B)
